# Supplementary material for: The SARS-CoV-2 nucleocapsid phosphoprotein forms mutually exclusive condensates with RNA and the membrane-associated M protein
Source: Nat Commun. 2021 Jan 21;12:502. doi: 10.1038/s41467-020-20768-y (PMC7820290; doi:10.1038/s41467-020-20768-y)
Supplement: Supplementary file 1 — Supplementary Information [file 41467_2020_20768_MOESM1_ESM.pdf]

Supplementary Information for:

The SARS-CoV-2 Nucleocapsid phosphoprotein forms mutually exclusive condensates with RNA and the membrane-associated M protein

Shan Lu, Qiaozhen Ye, Digvijay Singh, Yong Cao, Jolene K. Diedrich, John R. Yates III, Elizabeth Villa, Don W. Cleveland, Kevin D. Corbett

Supplementary Tables 1-3

Supplementary Figures 1-10

Supplementary Table 1. RNA sequences

All nucleotide numbers refer to NCBI RefSeq NC_045512.

RNA	Molecular Weight (Da)	Sequence
17-mer RNA	5740.4	AAGCAGCUAAGAGCGAA
UTR265<br>(nt 1-265)	86045	AUUAAAGGUUUAUACCUUCCCAGGUAACAAACCAACCAACUUUCGAUCUCUUGUAGAUCUGUUCUCUA<br>AACGAACUUUAAAAUCUGUGUGGCUGUCACUCGGCUGCAUGCUUAGUGCACUCACGCAGUAUAAUUAA<br>UAACUAAUUACUGUCGUUGACAGGACACGAGUAACUCGUCUAUCUUCUGCAGGCUGCUUACGGUUUCG<br>UCCGUGUUGCAGCCGAUCAUCAGCACAUCUAGGUUUCGUCCGGGUGUGACCGAAAGGUAAG
UTR1000<br>(nt 1-1000)	325393.5	AUUAAAGGUUUAUACCUUCCCAGGUAACAAACCAACCAACUUUCGAUCUCUUGUAGAUCUGUUCUCUA<br>AACGAACUUUAAAAUCUGUGUGGCUGUCACUCGGCUGCAUGCUUAGUGCACUCACGCAGUAUAAUUAA<br>UAACUAAUUACUGUCGUUGACAGGACACGAGUAACUCGUCUAUCUUCUGCAGGCUGCUUACGGUUUCG<br>UCCGUGUUGCAGCCGAUCAUCAGCACAUCUAGGUUUCGUCCGGGUGUGACCGAAAGGUAAGAUGGAGA<br>GCCUUGUCCCUGGUUUCAACGAGAAAACACACGUCCAACUCAGUUUGCCUGUUUUACAGGUUCGCGAC<br>GUGCUCGUACGUGGCUUUGGAGACUCCGUGGAGGAGGUCUUAUCAGAGGCACGUCAACAUCUUAAAGA<br>UGGCACUUGUGGCUUAGUAGAAGUUGAAAAAGGCGUUUUGCCUCAACUUGAACAGCCCUAUGUGUUCA<br>UCAAACGUUCGGAUGCUCGAACUGCACCUCAUGGUCAUGUUAUGGUUGAGCUGGUAGCAGAACUCGAA<br>GGCAUUCAGUACGGUCGUAGUGGUGAGACACUUGGUGUCCUUGUCCCUCAUGUGGGCGAAAUACCAGU<br>GGCUUACCGCAAGGUUCUUCUUCGUAAGAACGGUAAUAAAGGAGCUGGUGGCCAUAGUUACGGCGCCG<br>AUCUAAAGUCAUUUGACUUAGGCGACGAGCUUGGCACUGAUCCUUAUGAAGAUUUUCAAGAAAACUGG<br>AACACUAAACAUAGCAGUGGUGUUACCCGUGAACUCAUGCGUGAGCUUAACGGAGGGGCAUACACUCG<br>CUAUGUCGAUAACAACUUCUGUGGCCCUGAUGGCUACCCUCUUGAGUGCAUUAAAGACCUUCUAGCAC<br>GUGCUGGUAAAGCUUCAUGCACUUUGUCCGAACAACUGGACUUUAUUGACACUAAGAGGGGUGUAUAC<br>UGCUGCCGUGAACAUGAGCAUGAAAUUGCUUGGUACACGGAACGUUCU
PS576<br>(nt 19786-20361)	187974.5	GAGCUUUGGGCUAAGCGCAACAUUAAACCAGUACCAGAGGUGAAAAUACUCAAUAAUUUGGGUGUGGA<br>CAUUGCUGCUAAUACUGUGAUCUGGGACUACAAAAGAGAUGCUCCAGCACAUAUAUCUACUAUUGGUG<br>UUUGUUCUAUGACUGACAUAGCCAAGAAACCAACUGAAACGAUUUGUGCACCACUCACUGUCUUUUUU<br>GAUGGUAGAGUUGAUGGUCAAGUAGACUUAUUUAGAAAUGCCCGUAAUGGUGUUCUUAUUACAGAAGG<br>UAGUGUUAAAGGUUUACAACCAUCUGUAGGUCCCAAACAAGCUAGUCUUAAUGGAGUCACAUUAAUUG<br>GAGAAGCCGUAAAAACACAGUUCAAUUAUUAUAAGAAAGUUGAUGGUGUUGUCCAACAAUUACCUGAA<br>ACUUACUUUACUCAGAGUAGAAAUUUACAAGAAUUUAAACCCAGGAGUCAAAUGGAAAUUGAUUUCUU<br>AGAAUUAGCUAUGGAUGAAUUCAUUGAACGGUAUAAAUUAGAAGGCUAUGCCUUCGAACAUAUCGUUU<br>AUGGAGAUUUUAGUCAUAGUCAGUUAGGUGGU

Supplementary Table 2. Experimental conditions for crosslinking mass spectrometry

Condition	MS sample	BS3-d0/BS3-d4
UTR265 40 ng/µL	Group 1	with RNA/no RNA
	Group 2	with RNA/no RNA
	Group 3	no RNA/ with RNA
PS576 40 ng/µL	Group 4	with RNA/no RNA
	Group 5	with RNA/no RNA
	Group 6	no RNA/ with RNA
UTR265 160 ng/µL	Group 7	with RNA/no RNA
	Group 8	with RNA/no RNA
	Group 9	no RNA/ with RNA
no RNA	Group 10	no RNA/ no RNA

Supplementary Table 3. Primer sequences used in this study

Name	Sequence
N-BamHI-F	GAATTCACGCGTGGGCCCGGGATCCACCATGTCTGATAATGGACCCCAAAATC
N-XhoI-R	TGAACCTCCAACCGGTGACTCGAGGGCCTGAGTTGAGTCAGCA
N-SRdel-F	CAAAAGGCTTCTACGCAGAAGGGCCTGCTAGAATGGCTGGCAATGG
N-SRdel-R	CCATTGCCAGCCATTCTAGCAGGCCCTTCTGCGTAGAAGCCTTTTG
N-SA-F	CAGCACGTGCCCGCAACGCAGCAAGAAATGCAACTCCAGGCGCCGCAAGGGGAACTGCACCTGCTAGAATGGCTGGC
N-SA-R	CTTGCTGCGTTGCGGGCACGTGCTGCGGCACGTGCTGCGGCTTGAGCGCCGCCTCTGGCCCCTTCTGCGTAGAAGCC
N-SD-F	GATCGTGACGATGACCGTGATCGCAACGATGACAGAAATGACACTCCAGGCAGCAGTAGG
N-SD-R	CACGGTCATCGTCACGATCATCGGCTTGATCGCCGCCTCTGTCCCCTTCTGCGTAGAAGC
N-IDR-210-246-del-F	GTAGGGGAACTTCTCCTGCTAGAACTAAGAAATCTGCTGCTGAGG
N-IDR-210-246-del-R	CCTCAGCAGCAGATTTCTTAGTTCTAGCAGGAGAAGTTCCCCTAC
T7-UTR265-F	TAATACGACTCACTATAGGGATTAAAGGTTTATACCTTCCCAGG
UTR265-R	CTTACCTTTCGGTCACACCC
UTR1000-R	AGAACGTTCCGTGTACCAAG
T7-PAC576-F	TAATACGACTCACTATAGGGGAGCTTTGGGCTAAGCGCAACA
PAC576-R	ACCACCTAACTGACTATGAC
N_F2_v1	TACTTCCAATCCAATGCATCTGATAATGGACCCCAAAATCAG
N_F247_v1	TACTTCCAATCCAATGCAACTAAGAAATCTGCTGCTGAG
N_R364_v1	TTATCCACTTCCAATGTTATTATGGGAATGTTTTGTATGCGTCAA
N_R419_v1	TTATCCACTTCCAATGTTATTAGGCCTGAGTTGAGTCAGCAC
N_R174_v1	TTATCCACTTCCAATGTTATTATTCTGCGTAGAAGCCTTTTGGC
N_R246_v1	TTATCCACTTCCAATGTTATTAGACAGTTTGGCCTTGTTGTTGTTG
N_F269_v1	TACTTCCAATCCAATGCAAATGTAACACAAGCTTTCGGCAG
N_F365_v1	TACTTCCAATCCAATGCACCAACAGAGCCTAAAAAGGACAAAAAG
N_F49_v1	TACTTCCAATCCAATGCAACTGCGTCTTGGTTCACCGCTC
N_R209_v1	TTATCCACTTCCAATGTTATTATCTAGCAGGAGAAGTTCCC
N_F210_v1	TACTTCCAATCCAATGCAATGGCTGGCAATGGCGGTGATG
N_F2C_v1	TACTTCCAATCCAATGCATGCAGCGATAACGGCCCCCAAAAC
N_F175_v1	TACTTCCAATCCAATGCAGGGAGCAGAGGCGGCAGTCAA

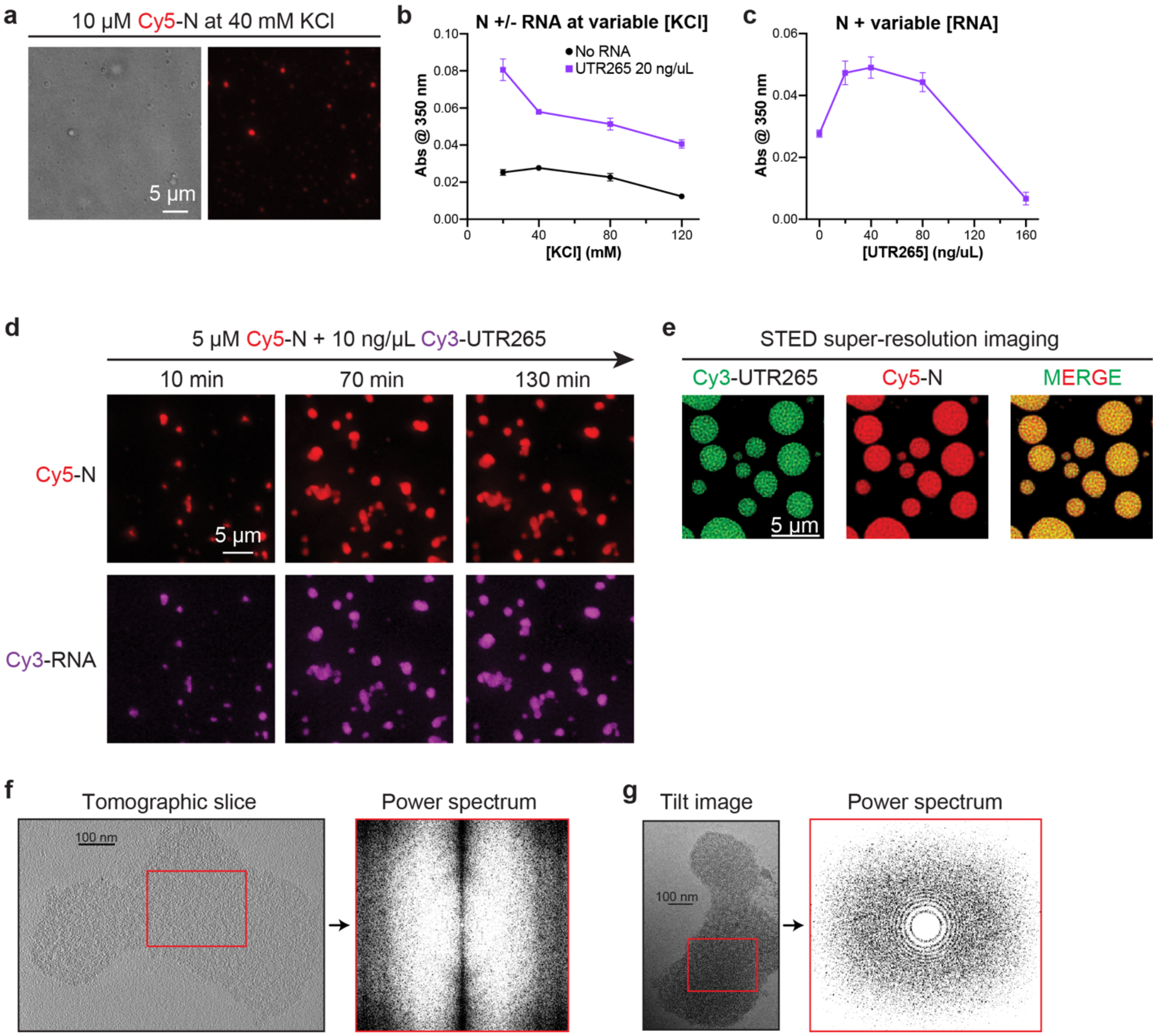

Supplementary Figure 1. Characterization of N protein+RNA condensates. (a) Example of condensate formation with isolated N protein. While the protein was isolated in high-salt buffers (1M NaCl) and showed the expected A_{260}/A_{280} ratio for pure protein, this phase separation behavior may be attributable to residual contaminating bacterial nucleic acids. Scale bar, 5 µm. (b) Turbidity analysis of N+RNA (UTR265) mixtures at different salt concentrations. Error bars represent standard deviation from 3 independent replicates. (c) Turbidity analysis of N+RNA (UTR265) mixtures at 40 mM KCl and different RNA concentrations. Error bars represent standard deviation from 3 independent replicates. (d) Time lapse imaging of N protein + UTR265 condensates. Scale bar, 5 µm. (e) STED super-resolution image of N+UTR265 condensates. Scale bar, 5 µm. (f-g) Power-spectrum analysis of selected regions (red) within a tomographic slice (f) or individual tilt image (g) of an N+RNA condensate. Thon rings are visible in the power spectrum of the tilt image due to the uncorrected contrast transfer function. The lack of other clear features in the power spectra indicates a lack of strong periodicity in the internal structure of the condensate.

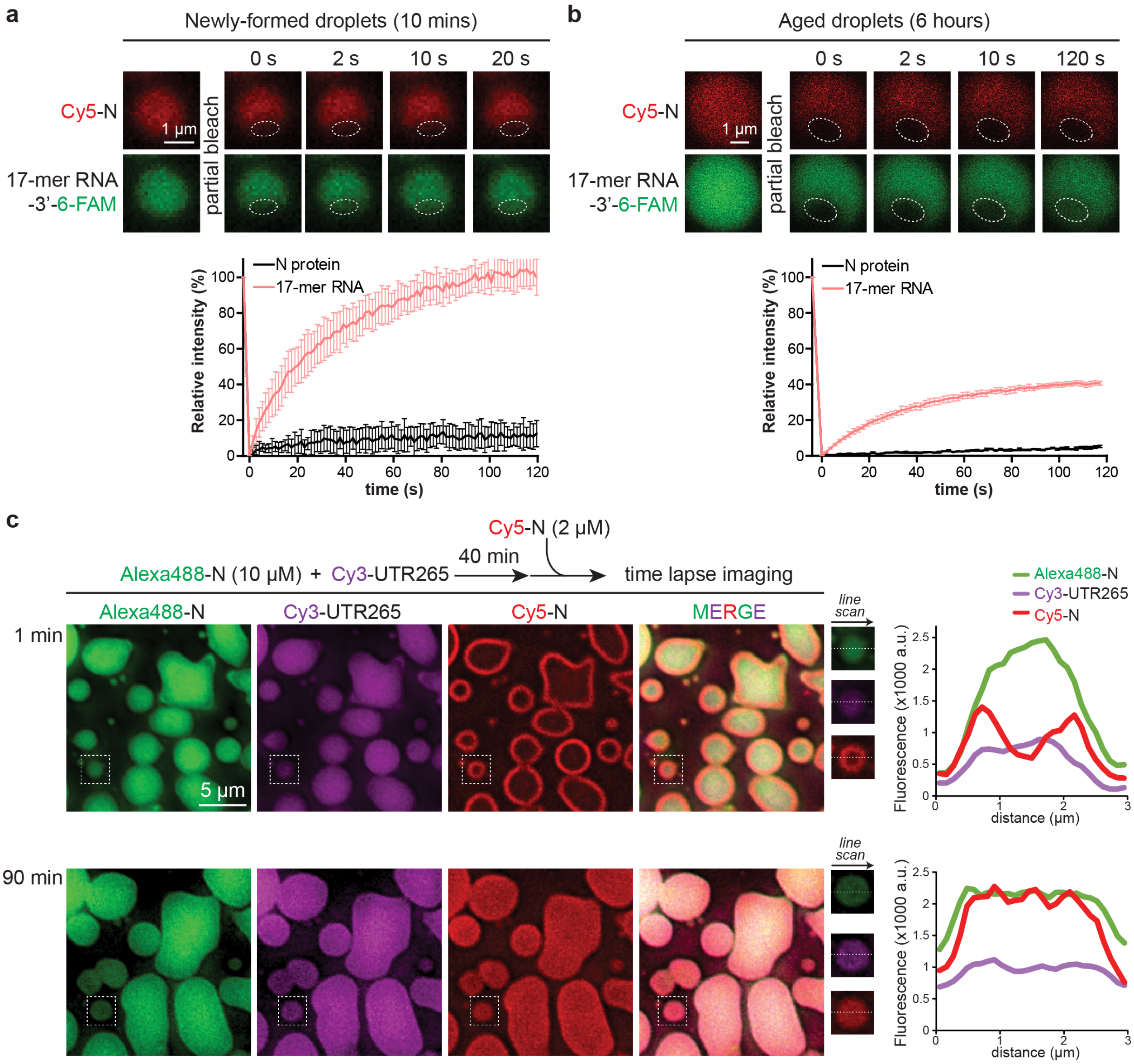

Supplementary Figure 2. Characterization of N+RNA condensates. (a) FRAP analysis of newly-formed condensates of 17-mer RNA + N protein. Scale bar, 1 µm. *Bottom:* Mean fluorescence intensity plot, with error bars representing standard deviation from FRAP analysis of 6 droplets. (b) FRAP analysis of aged condensates of 17-mer RNA + N protein. *Bottom:* Mean fluorescence intensity plot, with error bars representing standard deviation from FRAP analysis of 7 droplets. (c) Fluorescence images of the incorporation of new N protein into N+UTR265 protein condensates. N+UTR265 protein (10% Alexa488-labeled) condensates were preassembled for 40 min before adding 2 µM additions N protein (10% Cy5-labeled). The enlarged picture represents the localization of Alex488-N, UTR265, and Cy5-N in one condensate, 1 min or 90 mins after adding Cy5-N protein. *Right:* Fluorescence intensity plot Alexa488, Cy3 and Cy5 channels from line scans across the indicated condensate at 1 min and 90 mins.

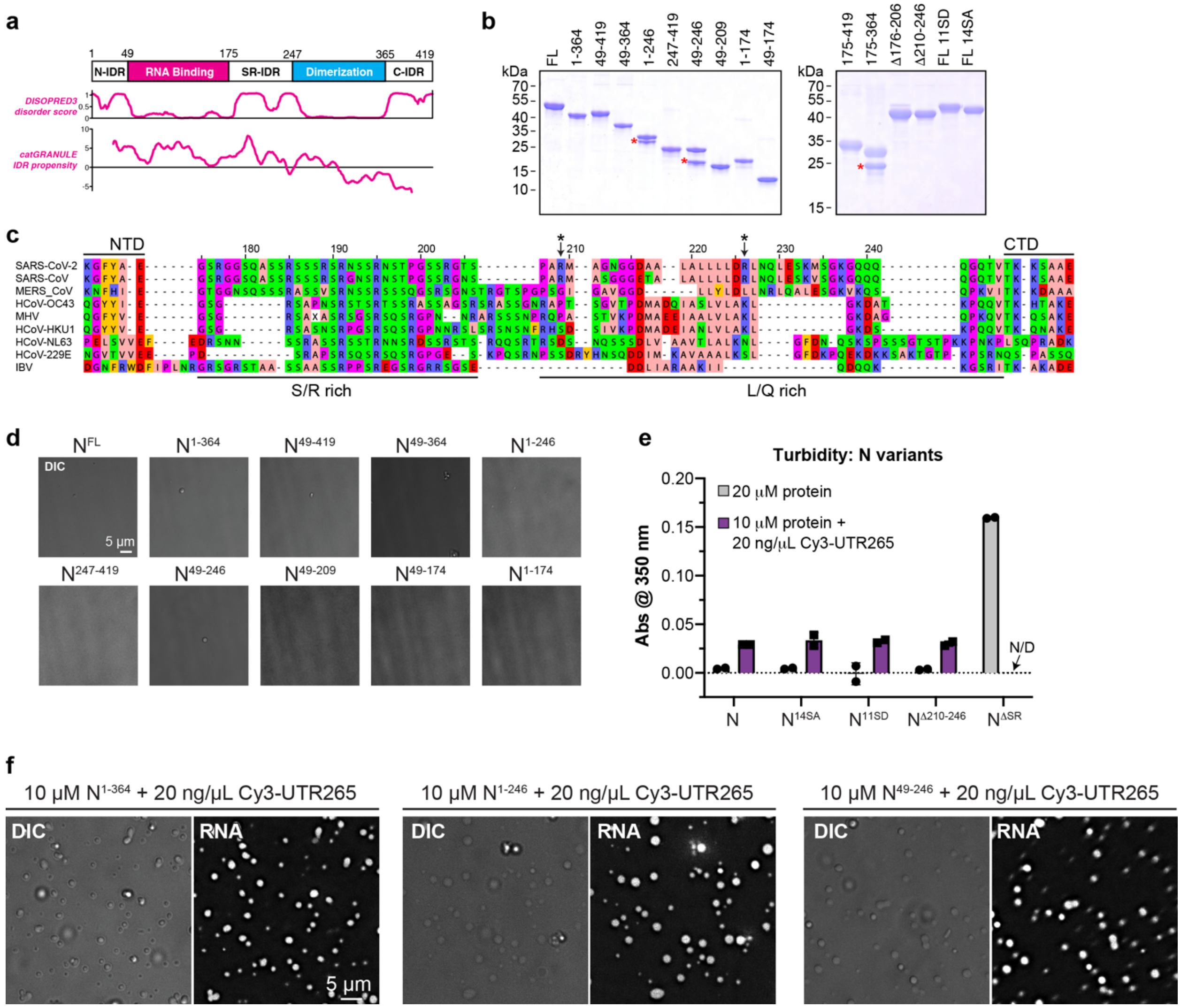

Supplementary Figure 3. Determination of domains involved in N+RNA phase separation. (a) N protein domains aligned with disorder propensity calculated by the DISOPRED3 server and catGRANULE IDR propensity score analysis. (b) SDS-PAGE analysis of all the N protein variants used for in vitro phase separation assay in **Figures 3 and 6**. Asterisks indicate products of proteolytic cleavage. (c) Sequence alignment of nine related coronavirus N proteins (SARS-CoV-2 NCBI Refseq ID QJA17760; SARS-CoV AYV99827; MERS-CoV QBM11755; HCoV-OC43 QBP84763; MHV AWB14620; HCoV-HKU1 ABG77571; HCoV-NL63 ABI20791; HCoV-229E AAA45463; IBV AAB24054), showing the central IDR. Asterisks indicate proteolytically sensitive sites identified by mass spectrometry. (d) DIC images of isolated N protein constructs. (e) Turbidity analysis of central IDR variants, in the absence of RNA (gray) and with UTR265 RNA (purple). Note that Cy3 has negligible absorbance at 350 nm. Turbidity of $N^{\Delta SR}$ was not determined with RNA due its tendency to aggregate even in the absence of RNA. (f) DIC and fluorescence images of phase separation behavior of N protein truncations $N^{1\text{-}364}$, $N^{1\text{-}246}$, $N^{49\text{-}246}$ when mixed with UTR265. Scale bar, 5 µm.

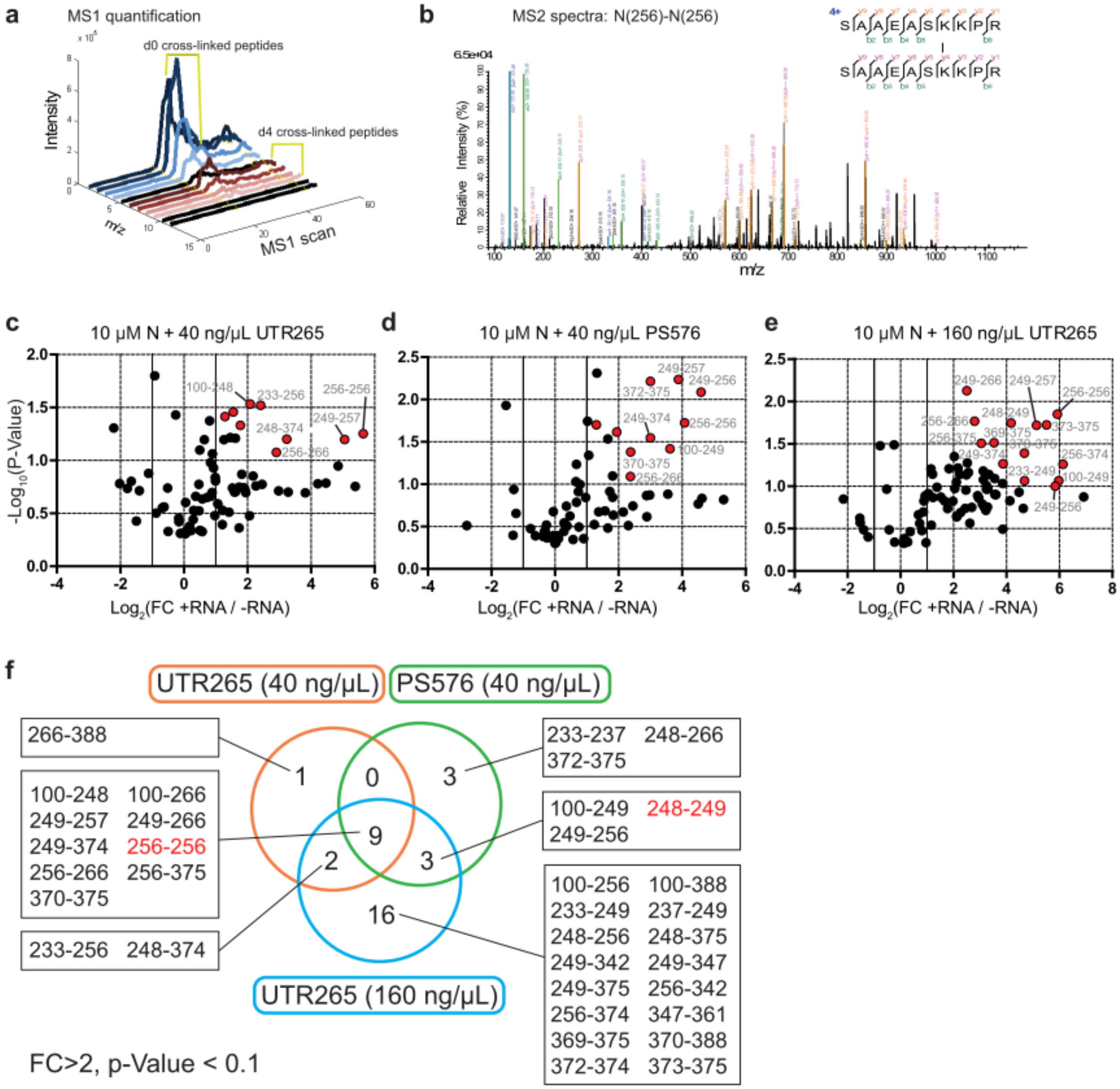

Supplementary Figure 4. Quantitative cross-linking mass spectrometry of N+RNA condensates. (a) Example MS1 spectra of cross-linked peptides. Matched fragment ions are labeled with the colors indicated in the sequence. (b) Extracted ion chromatogram of the cross-linked peptide in (a). (c-e) Volcano plots of identified cross-links in each experiment. (f) Venn diagram of crosslinks identified in the three conditions as in (c-e). Shown in red are two crosslinks that can be unequivocally assigned as intermolecular crosslinks. See **Supplementary Table 2** for details on experimental setup, and **Supplementary Data 1** for mass spectrometry results.

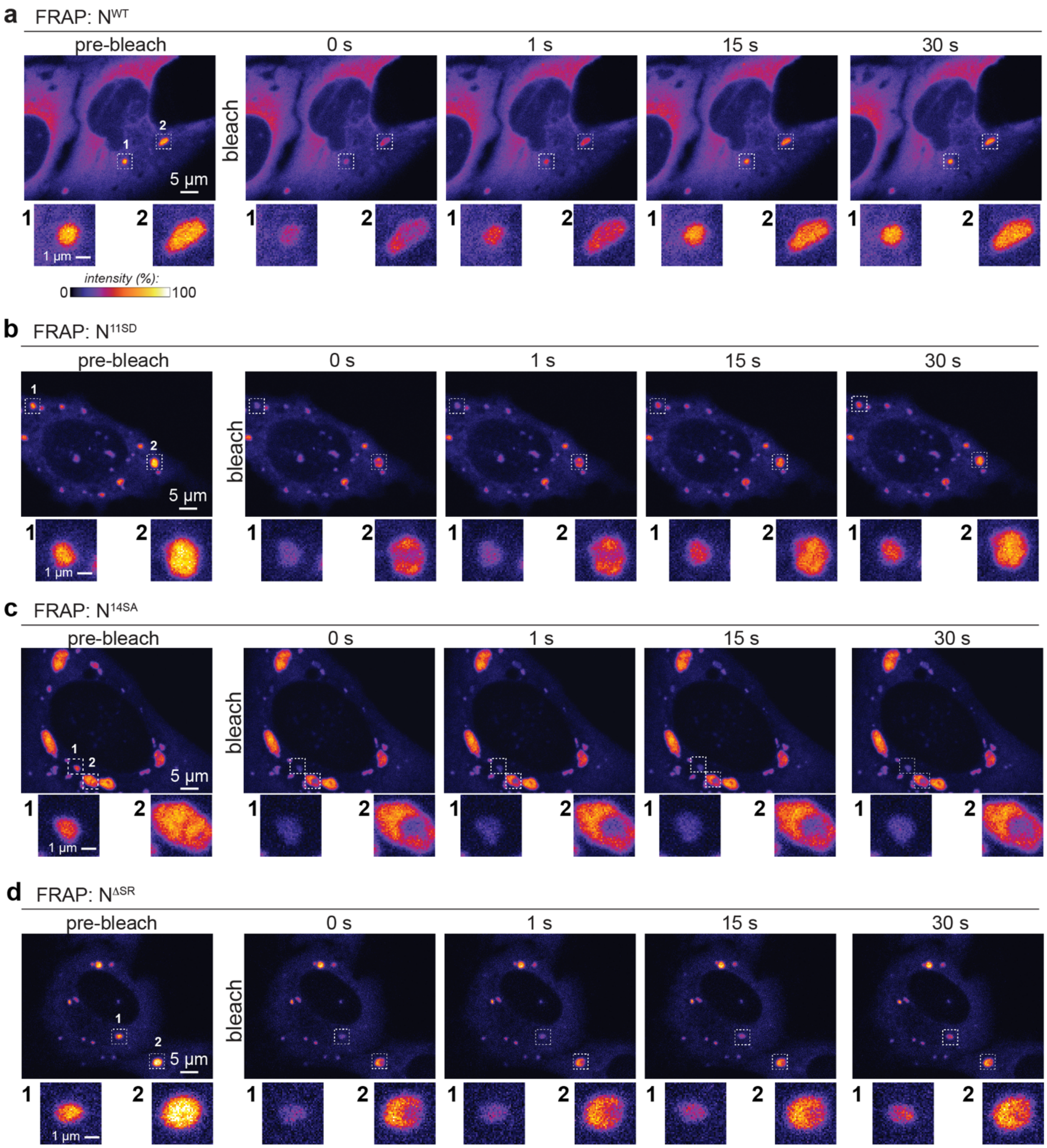

Supplementary Figure 5. FRAP analysis of N protein condensates in cells. Representative images from FRAP analysis of Clover-labeled N^{WT} (a, 7 droplets in 3 independent experiments), N^{11SD} (b, 9 droplets in 3 independent experiments), N^{14SA} (c, 13 droplets in in 3 independent experiments), and $N^{\Delta SR}$ (d, 10 droplets in 3 independent experiments). Enlarged pictures are the fluorescence images of one condensate after partial photobleaching (1) and one after full photobleaching (2). Scale bar, 5 µm for original image and 1 µm for enlarged images.

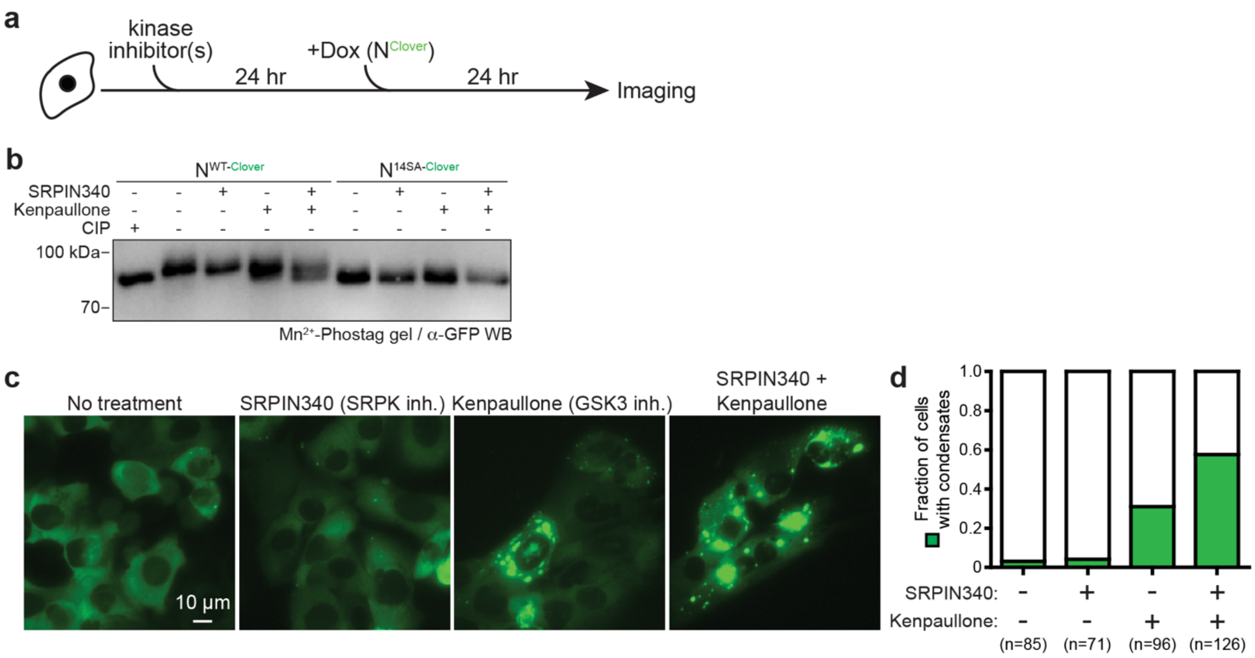

Supplementary Figure 6. N protein phosphorylation regulates condensation in cells. (a) Experimental scheme. Expression of N^{Clover} was induced 24 hours after addition of 10 µM kinase inhibitors (SRPIN340 or Kenpaullone), incubated a further 24 hours, then analyzed by Western blotting and imaging. (b) Western Blot showing dephosphorylation of N^{Clover} (wild-type or 14SA mutant) in the presence of kinase inhibitors. CIP: calf intestinal phosphatase. (c) Representative immunofluorescence images of untreated and kinase inhibitor-treated cells. (d) Fraction of cells showing N protein condensates when expressing Clover-tagged N in the presence and absence of kinase inhibitors (n represents the number of cells counted in each experiment).

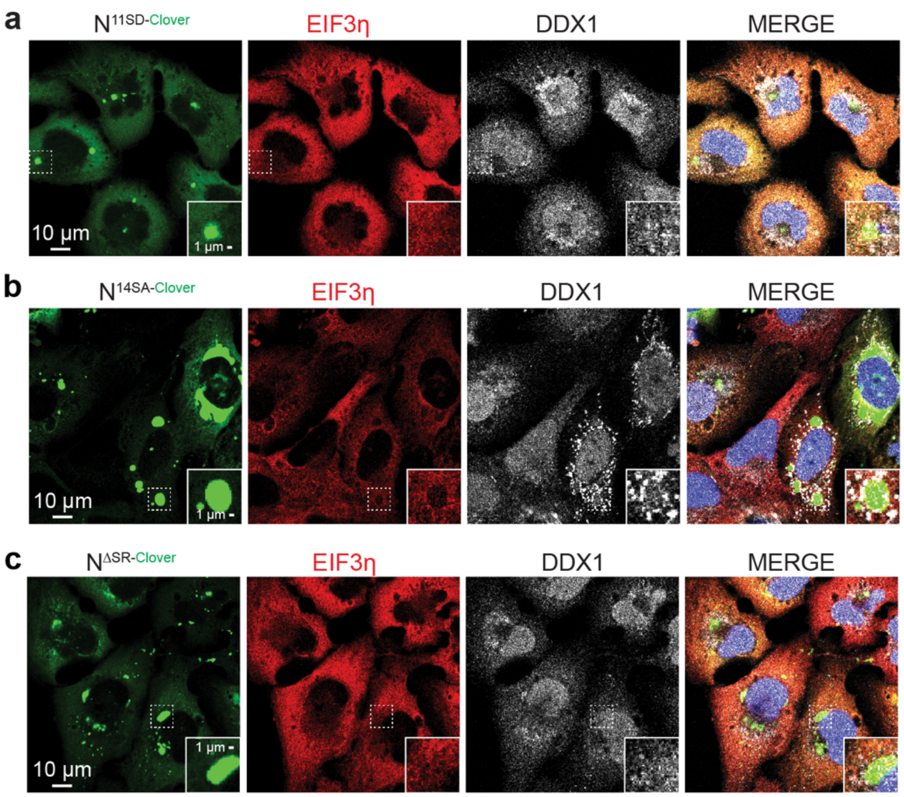

Supplementary Figure 7. Colocalization of N with stress granule proteins. (a-c) Representative images of U2OS cells expressing N^{11SD} (a), N^{14SA} (b), or $N^{\Delta SR}$ (c), comparing localization of N with G3BP1 and UBAP2L.

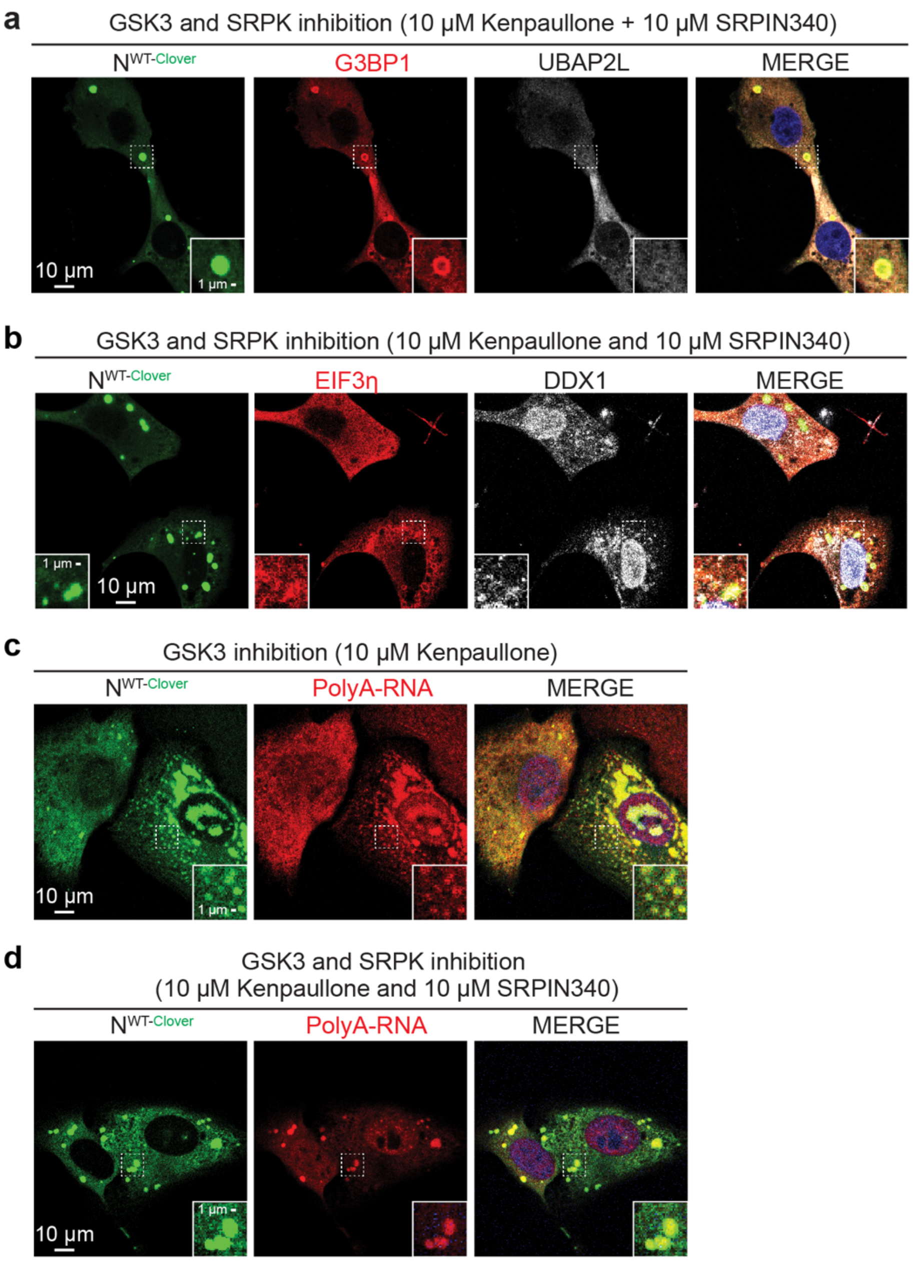

Supplementary Figure 8. Behavior of dephosphorylated N protein condensates. (a) Representative images of wild-type N^{Clover}, G3BP1, and UBAP2L in cells treated with 10 μM each Kenpaullone (GSK3 inhibitor) and SRPIN340 (SRPK inhibitor). (b) Representative images of wild-type N^{Clover}, EIF3η, and DDX1 in cells treated with Kenpaullone and SRPIN340. (c) Representative images of wild-type N^{Clover}, and PolyA-RNA (oligo-dT FISH) in cells treated with Kenpaullone. (d) Representative images of wild-type N^{Clover}, and PolyA-RNA in cells treated with Kenpaullone and SRPIN340.

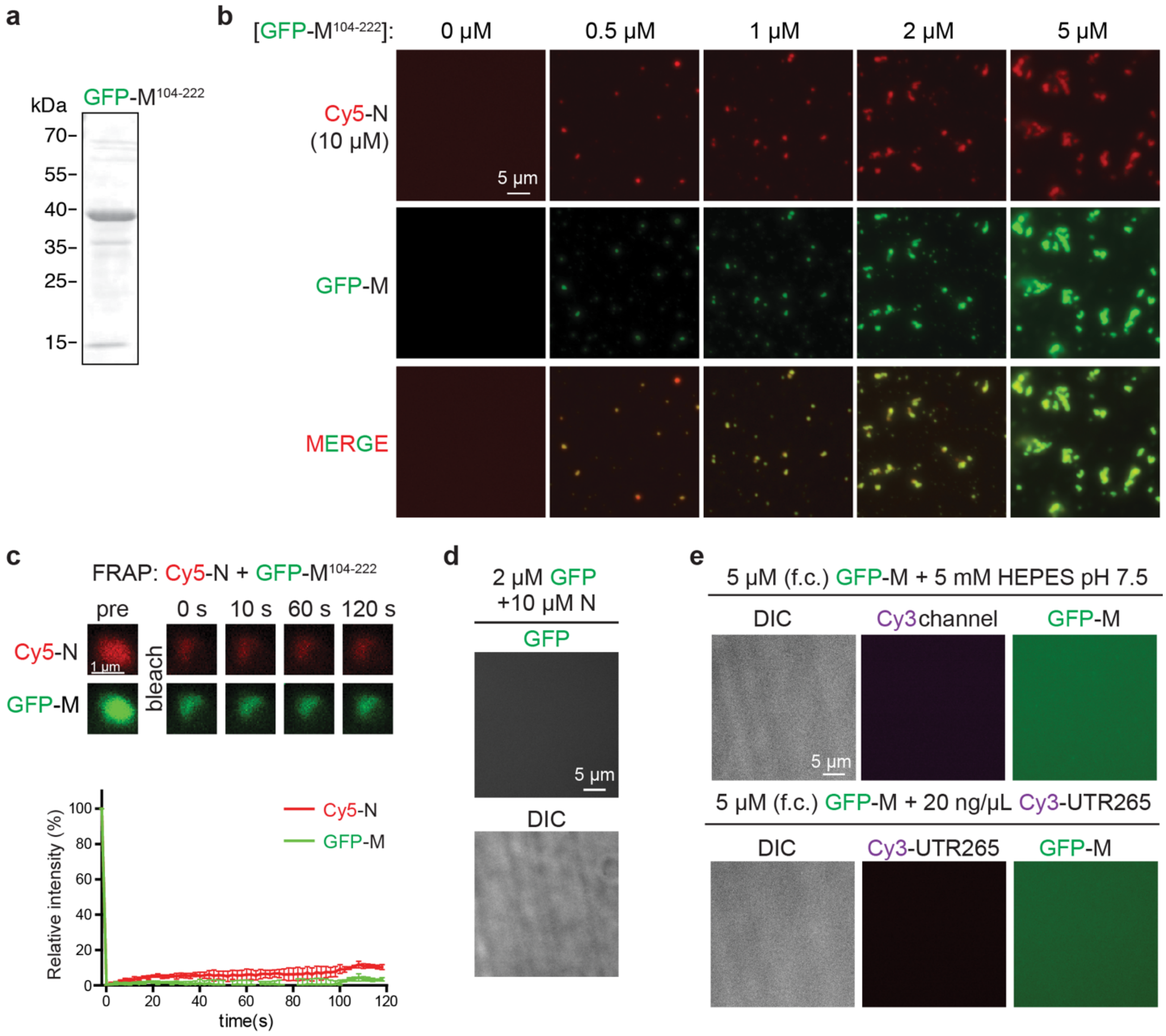

Supplementary Figure 9. Characterization of N+M condensates. (a) SDS-PAGE analysis of GFP-$M^{104\text{-}222}$. (b) Fluorescence images of phase separation of N protein when mixed with different concentration of GFP-$M^{104\text{-}222}$. N protein (10% Cy5-labeled) is used for this experiment. Scale bar, 5 µm. (c) *Top:* Representative images of a partial FRAP of N/M protein condensates. *Bottom:* Mean fluorescence intensity plot of N/M condensates in the FRAP experiment. Error bars represent standard deviation from FRAP analysis of 8 droplets. (d) Representative fluorescence and DIC images of 2 µM GFP mixed with 10 µM N protein. Scale bar, 5 µm. (e) Representative DIC and fluorescence images of GFP-$M^{104\text{-}222}$ when mixed with 20 ng/µL buffer (top) or Cy3-UTR265 (bottom). Images were taken 20 minutes after mixing. Scale bar, 5 µm.

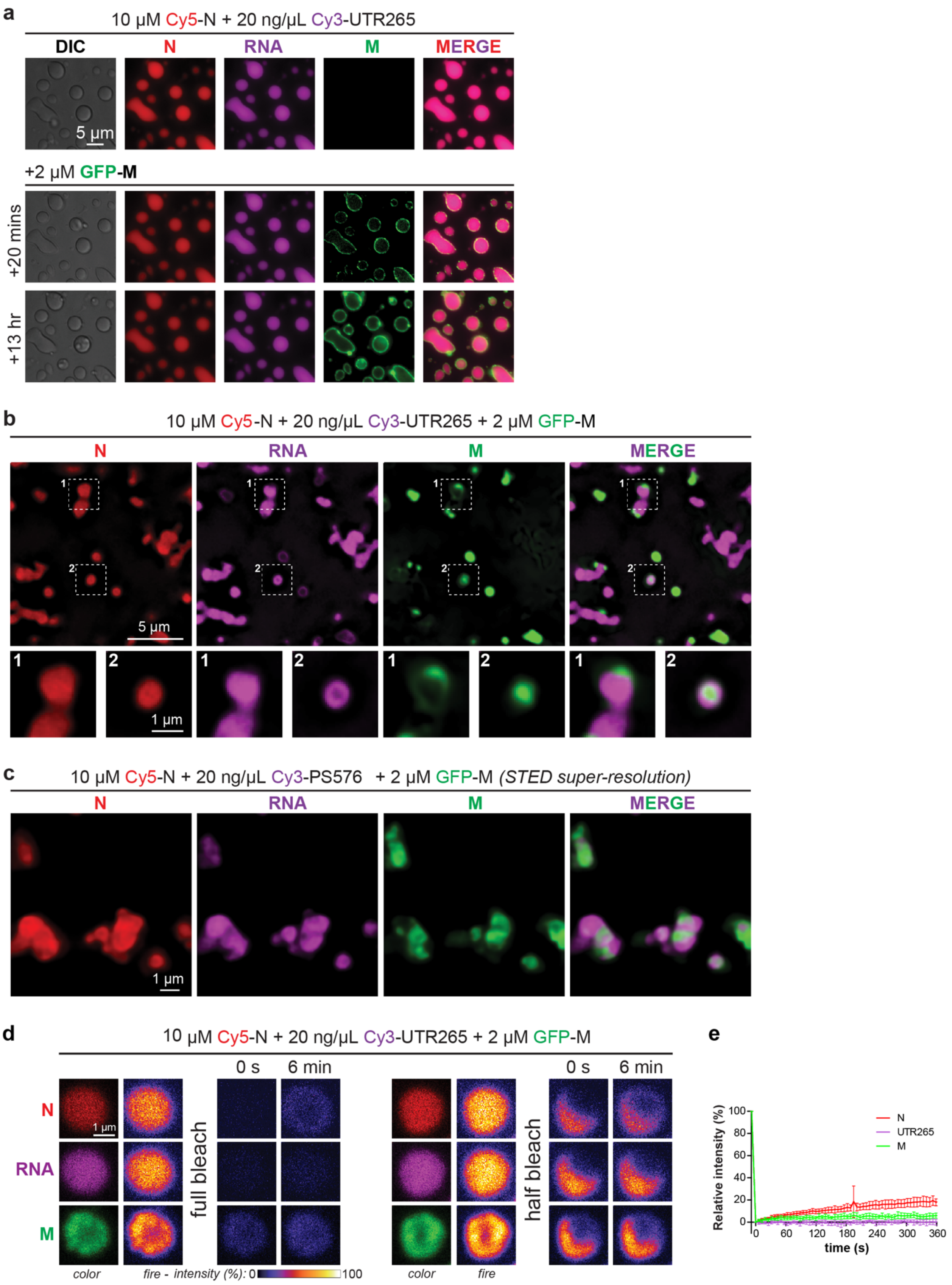

a
10 µM Cy5-N + 20 ng/µL Cy3-UTR265
DIC
N
RNA
M
MERGE
5 µm
+2 µM GFP-M
+20 mins
+13 hr
b
10 µM Cy5-N + 20 ng/µL Cy3-UTR265 + 2 µM GFP-M
N
RNA
M
MERGE
5 µm
1 µm
c
10 µM Cy5-N + 20 ng/µL Cy3-PS576 + 2 µM GFP-M (STED super-resolution)
N
RNA
M
MERGE
1 µm
d
10 µM Cy5-N + 20 ng/µL Cy3-UTR265 + 2 µM GFP-M
0 s
6 min
N
RNA
M
1 µm
full bleach
half bleach
color
fire - intensity (%): 0
100
color
fire
e
Relative intensity (%)
time (s)
N
UTR265
M

Supplementary Figure 10. M protein and RNA form mutually exclusive condensates with N protein. (a) Representative images of N+UTR265 condensates when mixed with GFP-$M^{104-222}$. Pre-assembled N+RNA condensates were formed for four hours and then 2 µM GFP-$M^{104-222}$ was added to the samples. Images were taken before and 20 min or 13 hours after adding M protein. Scale bar, 5 µm. (b) Representative images of condensates formed from mixing 10 µM N protein, 20 ng/µL UTR265 and 2 µM GFP-$M^{104-222}$. Enlarged images are example of two circumstances: 1: M protein forming a layer on the surface of N+UTR265 condensates; 2: RNA forming a layer on the surface of N+M condensates. Scale bar, 5 µm for original images and 1 µm for enlarged images. (c) Representative images of condensates formed from mixing 10 µM N protein, 20 ng/µL PS576 and 2 µM GFP-$M^{104-222}$. (d) FRAP analysis of 7 condensates of 10 µM N protein, 20 ng/µL UTR265 and 2 µM GFP-$M^{104-222}$. Pre-bleach images are shown in both color and fire mode (intensity/color scale shown below); post-bleach images are shown in fire mode. (e) Mean fluorescence intensity plot for FRAP analysis of N protein, UTR265 and M protein in N/UTR265/M condensates. Error bars represent standard deviation from FRAP analysis of 7 droplets.
