# Supplementary material for: The SARS-CoV-2 nucleocapsid phosphoprotein forms mutually exclusive condensates with RNA and the membrane-associated M protein
Source: Nat Commun. 2021 Jan 21;12:502. doi: 10.1038/s41467-020-20768-y (PMC7820290; doi:10.1038/s41467-020-20768-y)
Supplement: Supplementary file 3 — Description of Additional Supplementary Files [file 41467_2020_20768_MOESM3_ESM.pdf]

Description of Additional Supplementary Files

Supplementary Data 1: Quantification of cross-linked sites on N protein in the presence and absence of viral RNAs.
